# Thermoneutrality and Immunity: How Does Cold Stress Affect Disease?

**DOI:** 10.3389/fimmu.2020.588387

**Published:** 2020-11-20

**Authors:** Fiorella Vialard, Martin Olivier

**Affiliations:** Department of Microbiology and Immunology, Program in Infectious Diseases and Immunity in Global Health, The Research Institute of the McGill University Health Centre, McGill University, Montreal, QC, Canada

**Keywords:** thermoneutrality, murine model, immune functions, infectious diseases, metabolism, body temperature, immunity

## Abstract

One of the major challenges the scientific community faces today is the lack of translational data generated from mouse trials for human health application. Housing temperature-dependent chronic cold stress in laboratory rodents is one of the key factors contributing to lack of translatability because it reveals major metabolic differences between humans and rodents. While humans tend to operate at temperatures within their thermoneutral zone, most laboratory rodents are housed at temperatures below this zone and have an increased energy demand to generate heat. This has an impact on the immune system of mice and thus affects results obtained using murine models of human diseases. A limited number of studies and reviews have shown that results obtained on mice housed at thermoneutrality were different from those obtained from mice housed in traditional housing conditions. Most of those studies, focused on obesity and cancer, found that housing mice at thermoneutrality changed the outcomes of the diseases negatively and positively, respectively. In this review, we describe how thermoneutrality impacts the immune system of rodents generally and in the context of different disease models. We show that thermoneutrality exacerbates cardiovascular and auto-immune diseases; alleviates asthma and Alzheimer’s disease; and, changes gut microbiome populations. We also show that thermoneutrality can have exacerbating or alleviating effects on the outcome of infectious diseases. Thus, we join the call of others in this field to urge researchers to refine murine models of disease and increase their translational capacity by considering housing at thermoneutrality for trials involving rodents.

## Introduction

Thermoneutrality (TN) has been defined as the metabolic state of an organism in an environmental temperature at which it does not have to generate or lose heat (1). It is a relatively recent term in the scientific literature with the first reference dating to 1968 (2). The general interest in this concept is still under the radar. Despite this, TN is an important concept to consider when conducting research using one of the most common human disease models: the mouse (1, 3–7). Mice are frequently used in research because of ease in maintenance and genetic manipulation (3, 8). However, much of the research conducted on mice models does not translate well to important impact in human clinical trials due to differences in data reporting; study set-up parameters; and, mouse *vs*. human metabolism (9–11). A substantial improvement and refining of murine models is required in order to reduce the variability of findings due to metabolic differences (11). Some problems in metabolic reproducibility stem from circadian rhythm differences with humans; social stressors; and, facility-based differences in the gut microbiome, but another important factor is housing temperature (5, 6, 11–16). In the laboratory setting, mice are routinely housed at temperatures (20–22°C) below their thermoneutral zone (TNZ) (29–34°C) (5). In this review, we will refer to the standard temperature used in most research settings as sub-optimal temperature (ST) because it subjects mice to a constant metabolic stress (5). Rodents are particularly affected by exposure to ST because they rely heavily on non-shivering thermogenesis for heat generation and possess a greater thermogenic demand due to their greater surface area to volume (SA/V) ratio than other animals (1, 5). Consequently, mice lose heat more quickly and need more energy to maintain their body core temperature (BCT) (3). Behavioral signs of cold stress can be observed in traditional housing systems: mice tend to huddle together or use nesting material in order to prevent heat loss (17). Mice pups also vocalize more to indicate distress when they are housed below their thermoneutral temperature (TT) (18). In addition, given the choice, mice preferentially choose an area in their housing that is closer to their TT, regardless of the availability of nesting material (1, 19). The goal of this review is to present a thorough overview of what is currently known about TN and its effect on murine models of disease.

## Role of Environmental Temperature on Mouse Metabolism

**Figure 1:**
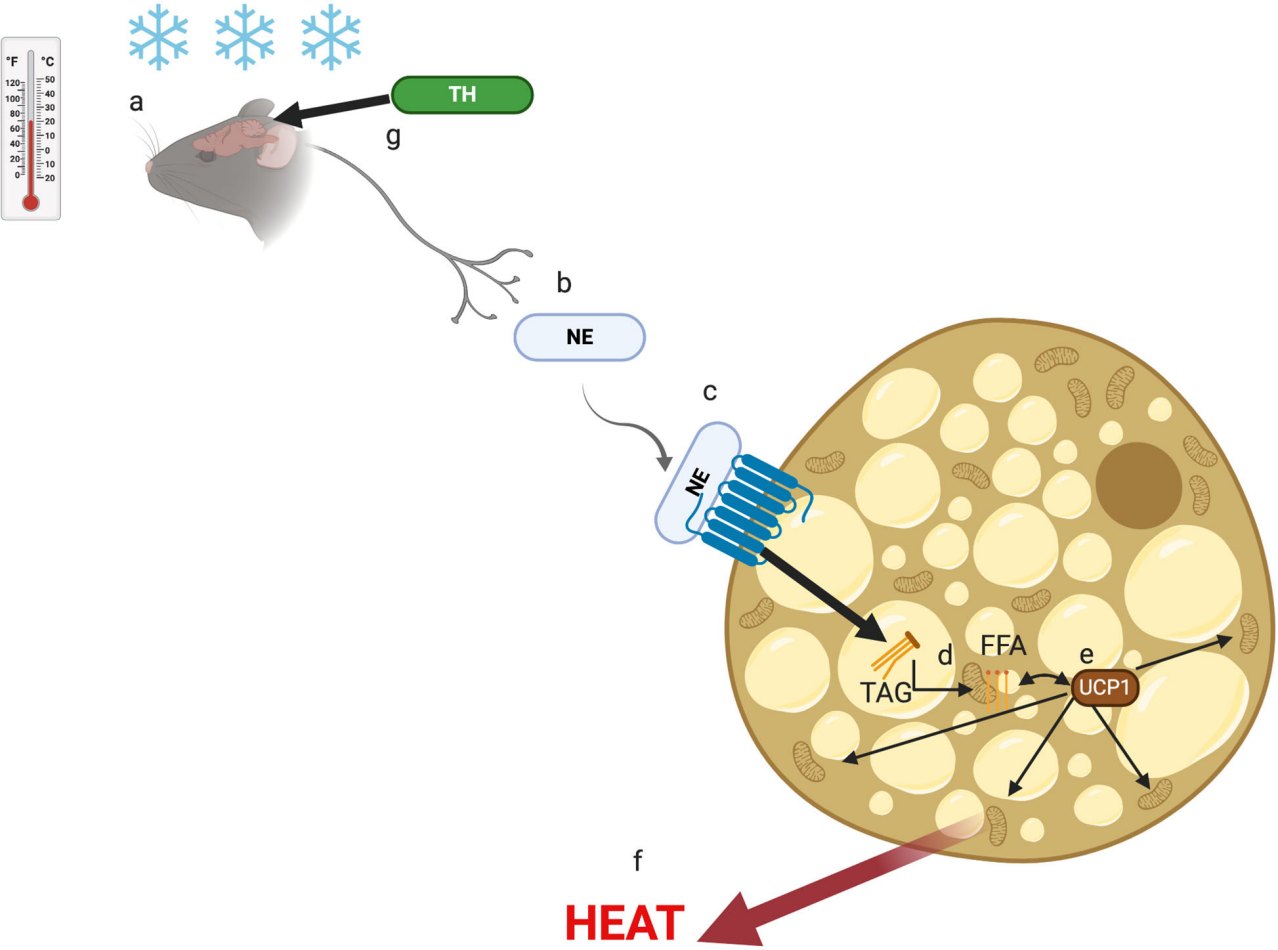
The non-shivering thermogenesis activation pathway. Cold stress is sensed by the brain **(A)** and signals the sympathetic nervous system to release norepinephrine (NE) **(B)** that interact with b-adrenergic receptor (b-AR) on brown adipocytes **(C)**. As a result, triglycerides (TAG) are broken down to free fatty acids (FFA) **(D)** that interact with uncoupling protein-1 (UCP-1) **(E)** to induce the mitochondria to generate heat **(E)**. The thyroid hormone (TH) plays a role in the cold stress **(F)** sensor region of the brain **(G)**.

The rodent response to cold stress through brown adipose tissue (BAT)-mediated non-shivering thermogenesis is well understood (11). The environmental ST sensed by the brain (**Figure 1A**) causes the release of norepinephrine (NE) from the sympathetic nervous system (**Figure 1B**) (11). The released NE interacts with β-adrenergic receptors (β-AR) on the surface of brown adipocytes (**Figure 1C**) (11), resulting in break-down of triglycerides (TG) within the BAT into free-fatty acids (FFA) (**Figure 1D**). The FFA interact with uncoupling protein-1 (UCP-1) present in the cytosol (**Figure 1E**) of BAT cells and stimulate the mitochondria to produce energy in the form of heat (**Figure 1F**) (11). The thyroid hormone (TH) is thought to play a role in this metabolic process by stimulating the brain regions responsible for BAT activation (**Figure 1G**) (11).

Various physiological and metabolic effects of housing mice at TT compared to ST have been documented in a number of publications (20–29). At TT, mice have lower energy expenditure in light and dark cycles; higher energy efficiency; and decreased nutrient mobilization and metabolization due to the removal of the cold stressor (21–24). They also spend less time awake, suggesting that mice housed at ST experience sleep deprivation (3, 22). Mice housed at TT exhibit less cancellous bone loss and are better adapted to deal with metabolic stressors such as sleep deprivation or heat stress (20, 26, 27, 30). Most mouse models of longevity are more resistant to ST due to their lower BCT (3). In addition, the effects observed on mice housed at TT can often be mimicked at ST by treating the mice with β-AR antagonists, which suppresses the NE pathway for heat generation (27–29).

## Role of Environmental Temperature on the Immune System

**Figure 2:**
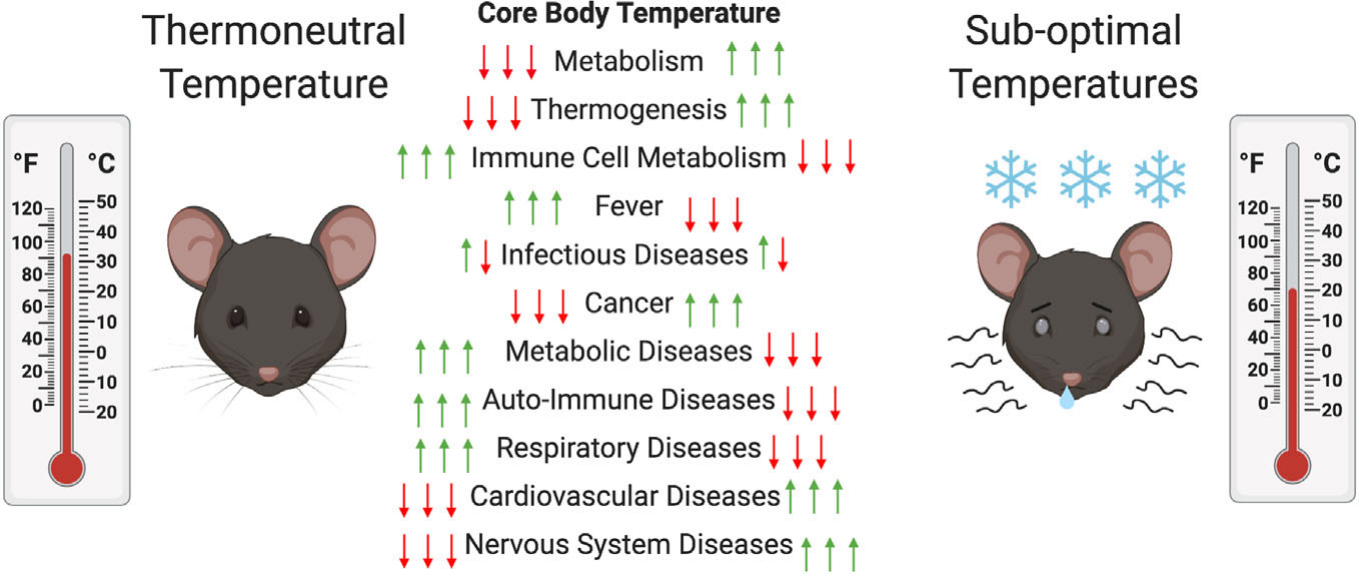
Changes in mouse metabolism and diseases at thermoneutral (TT) compared to suboptimal temperatures (ST). In order to maintain their core body temperatures (CBT) mice have to increase and decrease their metabolic activity to increase or decrease thermogenesis at ST compared to TT, respectively. This can decrease or increase the metabolic activity of their immune cells and have alleviating (red arrow) or exacerbating (green arrow) effects on various disease models. In the case of infectious diseases both can occur depending on the type of pathogen.

Another major consequence of ST housing is decreased energy availability for mice immune system. Energy needed to drive immune processes is instead redirected toward heat generation (8), resulting in clear differences in the immune response of mice at ST compared to TT. Some studies have reported an increase in thermogenesis-associated M2 macrophages at ST (31). These macrophages are associated with adipose tissue and provide the same function as NE during thermogenesis (31). Other studies have reported an increase in BAT inflammation with production of pro-inflammatory cytokines (MCP1, IFNγ, TNFα, IL-1β, and IL-6) during exposure to ST (32). These alternatively activated macrophages have been shown to be associated with expression of IL-4 and IL-13 and are involved in the expression of catecholamines by BAT (33). T cell proliferation has been shown to be suppressed in part by myeloid-derived suppressor cells (MDSCs) that are generated in greater number *via* β-AR signaling under ST conditions (34, 35). Another study showed that BAT inflammation at ST is mediated by the type 2 immune response in mice (36). Treatment of mice at TT with IL-33, which triggers ILC2s that are involved in the type-2 response, resulted in an increase in BAT size, activity, and energy expenditure (36). In addition, mice and rats housed at ST tend to experience latent hypothermia after immune challenge with LPS, while those at TT undergo a fever response (37, 38). This has also been documented in other animals (e.g., pigs and cattle) (39). In rats, the ST-hypothermic response is age-dependent, with a reduced effect observed in older animals (40, 41). At TT, a reduction in activity is generally sufficient to fight an immune challenge compared to the need for increases in processes such as oxygen consumption; blood glucose metabolism; and, FA metabolism at ST (42). Some immune factors, such as TNF-α, Il-6, and Il-1β, have been implicated in the differential response to immune challenge at TT compared to ST due to their function as pyrogens in the fever response (27, 41, 43). In addition, high mobility group box 1 (HMGB1), the macrophage-derived factor involved in tissue repair and tissue fibrosis, was found at a higher level in circulation at TT and higher liver inflammation was observed (44). Interestingly, immune-challenged rodents tend to prefer environmental temperatures closer to their TT, indicating that TT is beneficial in the response to various pathogens (40). However, in this review we will outline cases in which housing at TT can exacerbate disease presentation (see **Table 1**). **Figure 2** outlines the effect of thermoneutrality on different models of disease.

**Table 1:** Effects of sub-optimal *vs*. thermoneutral environmental temperatures on the presentation of murine disease models.

Mouse model	ST (20–24°C)	TT (27–36°C)
Obesity	• Alleviated (45, 46) • Lower glucose intolerance (45, 46) • Resistance to treatment (47, 48)	• Exacerbated (45, 46) • Greater glucose intolerance (45, 46) • HFD, UCP-1 KO, and T2D KO mice (45, 49, 50) • Better responses to treatments (47, 48)
Cardiovascular diseases (atherosclerosis)	Alleviated (44, 51)	Exacerbated • Increased markers (i.e., plaque build-up and immune cell infiltration) (44, 51)
Non-alcoholic fatty liver disease	Female resistance (52)	Sex-independent establishment (52)
Microbiome	Remodeling of the small intestine (53)	• Increased intestine permeability (52) • Increase in gram neg. bacterial populations (52)
Cancer	• Suppressed cytotoxic activity (34, 54, 55) • Higher anti-apoptotic activity (25) • Resistance to treatment (25, 56, 57)	• Tumor resistance (4, 34, 54, 55, 57–59) • Improved response to treatment (25, 56, 57)
Viral infections	Exacerbated (20, 60, 61)	• Alleviated (rabies, influenza, and Coxsackie virus) (20, 60, 61) • Improved immunization (Coxsackie and foot and mouth disease virus) (61, 62)
Bacterial infections	Hypothermia response (63)	• Fever response (63) • Exacerbation of systemic inflammatory response (*E. coli*) (63, 64) • Lower mortality in *T. rickettsia* infection (65) • Improved immunization against *F. tularensis* (66)
Parasitic infections	• Increase in parasitemia and decrease in schizont size (*P. berghei*) (67, 68) • Nodular presentation *L. mexicana* (69)	• Higher mortality rates of *P. berghei* infection (70) • Protective against *T. cruzi* infection (71, 72) • Cutaneous presentation *L. mexicana* (69)
Auto-immune diseases (graft *vs*. host disease)	Resistance (28, 35)	Exacerbated response (28, 35)
Respiratory diseases (asthma)	Exacerbated (73)	Alleviated (73)
Nervous system diseases (Alzheimer’s disease)	Increased sensitivity of 3×Tg-AD mouse model (74)	Improved memory function (74)

## Respiratory Diseases 

The effect of TN on the respiratory system of mice has only been investigated in the context of the ovalbumin-induced asthma disease model (73). Liao et al. observed lower inflammatory cell counts driven by a decrease in eosinophil populations in bronchoalveolar lavage from these mice (73). The airway hyperresponsiveness to asthma was also reduced in mice at TT compared to ST, characterized by lower IL-4, IL-10, and IgGE levels and higher IFNγ levels (73). These results were attributed to an increase in regulatory T cell infiltration in the lungs at the higher temperature, which dampens the inflammatory response (73). In this context, TN mediates a protective immune response by promoting tight regulation of immune cells at the site of inflammation compared to ST. However, this phenomenon should be tested on other respiratory disease models.

## Graft *vs*. Host disease

Another disease in which TN housing has revealed a gap in the understanding of murine models is in the context of graft *vs*. host disease (GVHD) (28, 35). This disease occurs when the immune system of an individual grafted with cells or tissue from a genetically distinct individual recognizes it as foreign and induces a fulgurant immune response. It was previously thought that congenic mice were resistant to the establishment of GVHD but Leigh et al. (27) found that grafting mice with bone marrow-derived T cells caused severe GVHD characterized by lesions in the intestines and liver of mice housed at TT (28). They attributed the resistance in mice housed at ST to high NE levels that impaired their immune response. Resistance at ST was reversed when the mice were treated with a β-AR antagonist or the gene encoding β-AR was knocked-out (28). Contrarily, when mice were treated with a β-AR at TT agonist, GVHD was decreased (28). In another study, the same group demonstrated that mice with T cells lacking β-AR housed at ST experienced a Th1 response when they were implanted with an allo-graft, suggesting that β-AR signaling plays a direct role in the severity of GVHD (35). They also showed that treating mice with a β-AR agonist decreased the severity of GVHD (35). These two studies demonstrated a clear link between TN and a metabolically activated immune system mediated by β-AR signaling.

## Bacterial Infections

In the context of the fever-response to bacterial agents, housing temperatures closer to TN may alleviate or exacerbate disease presentation. As mentioned previously, rodents challenged with LPS develop hypothermia when housed at ST and fever when housed at TT (63). This was also observed in rats treated with a dose of *Escherichia coli* which induced a systemic inflammatory response syndrome (63). Hypothermic rats housed at ST also had decreased neutrophil infiltration in lungs; lower arterial pressure; decreased endotoxemia; increased liver bacterial load; lower organ dysfunction; and, lower mortality rates (63). This demonstrates that fever in rodents housed at TT can exacerbate disease, probably due to increased sensitivity to infectious agents and immune-cell mediated bacteriolysis followed by sepsis (63, 64). Interestingly, changes were not seen in levels of the major fever-associated cytokines (63).

It is important to note that an exacerbated response does not occur in all bacterial infections. Mice injected with *Typhus rickettsia*, which develops more favorably in a colder environment, had a 75% decrease in mortality rates at TT compared to ST (65). Importantly, increased immune sensitivity at TT can be beneficial in vaccination against bacterial disease. Balb/c mice housed at TT vaccinated with live attenuated *Francisella tularensis* had a higher IFNγ-mediated antigen-specific T-cell response when challenged with the pathogen (66). Unfortunately, information on the effect of TT on bacterial diseases in mice is very limited. The studies cited above show variation in the bacterial species or strain; animal species, strain, age, and sex; acclimatization time; type of cage system; and, type of inoculum tested (63, 65, 66). This makes it difficult to draw solid conclusions pertaining to the specific role of TN in the context of bacterial infections.

## Viral Infections

A TN environment may also be protective for the murine host experiencing viral infection. Lower inflammation (i.e., lower leucopenia and lower cytokine induction) was reported in mice infected with influenza virus at TT compared to ST, despite the presence of equivalent viral titers in both groups (20). The animals at TT also exhibited a fever-response and better sleep recuperation (20). It has also been shown that mice infected with rabies virus at 37°C had lower mortality rates and lower brain-viral burden than those at 24°C (60). Similar results were obtained with mice infected with Coxsackie virus at 36°C compared to 25°C (61). In addition, mice housed at 36°C responded better to a pre-challenge live-vaccine (61). Interestingly, the effect observed at TT was absent in animals treated with an immune-suppressor, suggesting that the change in survival and tissue-burden was mediated by increased immune cell competence at TT (61). In another study, mice at TT that received a foot-and-mouth DNA vaccine had a superior immune response, with increased IgG levels, T cell proliferation, IFNγ production in both CD4+ and CD8+ T cells, and IL-4 production in CD4+ T cells, compared to heat-stressed mice at 38°C (62). However, this response was not tested at ST and the temperature defined by researchers as TT (24°C) was lower than the generally recognized TN range for mice (5, 62). There may be viral infections in which TN is detrimental for the host, but these have not been reported in the literature.

There are important limitations in the investigations of the TN effect on the response to viral infections. The first is the wide range of temperatures defined as being thermoneutral (20, 62). In addition, as with studies on bacterial infections, mouse strain, age, sex, and acclimatization time should be taken into account in future studies. Use of additional viral disease models will be required to gain a better understanding of TN on the immune response to viruses.

## Parasite Infections

The effects of TN on the outcome of parasitic infections are still largely unraveled. However, a few studies investigating the role of temperature on parasitized murine hosts offer insights into potential consequences of TN housing on parasitic disease models (67–72).

Malaria-causing parasites belonging to the *Plasmodium* genus have a complex life cycle (75). The species that infect mammals incorporate temperature changes that occur during transmission between invertebrate to vertebrate hosts into their development (75). The blood-stage of these parasites causes the majority of malaria-associated symptoms, including fever, which also has consequences on the parasite (75). Thus, housing *Plasmodium-*infected mice at TT could have significantly different clinical presentation compared to those housed at ST. There is some evidence to support this possibility in the literature. For example, mice exposed to cold temperatures had an increase in parasitemia after 4 days, suggesting that parasitemia may diminish at TT (67). In addition, blood-stage parasite development may be affected by TT. Rats infected with two different strains of *Plasmodium berghei* had schizont-stage parasites of smaller sizes when exposed to colder temperatures (12°C) compared to TT, highlighting a potential protective effect of lower temperatures (68).This difference was not observed in rats housed at 20°C (68). Housing density also seems to be a factor in the outcome of *Plasmodium* infection. Mice housed in groups of five at TT (27°C) had higher mortality rates than those housed individually. The latter had the same mortality rates as mice housed at ST (21°C) in groups of five, but higher rates than those housed individually at 21°C (70). The study investigators concluded that the group-housed mice were probably more susceptible to disease at both temperatures because of their inability to dissipate body heat during the fever-response induced by the blood-stage of malaria (70). In this context, TT would be expected to exacerbate the malarial symptoms. In order to gain a better understanding of TN and malarial disease, other aspects of this disease such as its role on sequestration or spleen megalopathy should be investigated.

It is unclear whether malarial disease clinical signs would be diminished or exacerbated at TN. However, higher temperatures seem to have a protective effect against infections with *Trypanosoma cruzi*, the causative agent of Chagas disease. Mice infected with the parasite at 35°C survived and overcame the infection, whereas those infected at 26°C experienced chronic infections (71). Similar results were obtained in a different mouse strain, where a reduction of parasitemia and tissue pathology, and an increase in immune responsiveness was observed (72). In both studies, the protective effects of high temperatures were completely reversed by treatment with immunosuppressors or depletion of CD8+ immune cells, implicating the competence of immune cells at TT (71, 72).

Finally, the environmental temperature of the host has been tested on an infection caused by a third type of parasite: *Leishmania mexicana* (69). Balb/C mice at 32°C had inoculation site nodular inflammation with macrophage infiltration and phagocytosis presentation whereas those at 22°C had a more cutaneous presentation with mast cell infiltration (69). Both showed visceralization as confirmed by parasite culture from lesions, immunohistology, and PCR (69). However, the mice were not conditioned to the different temperatures beforehand (69).

The parasitic infections described above only represent a minor fraction of all parasitic diseases that could be impacted by TT. For each of those diseases, a wide variety of models exist, constituted of various mouse strain and parasite species/strain combinations that can be investigated at TN. In addition, many of the studies described above did not acclimatize the animals to the temperature conditions, which could bias the results (68, 69, 72). Murine host sex and age differences, as well as difference in acclimatization time and caging system will also have to be taken into consideration when conducting future experiments.

## Cancer

TN has been investigated more frequently in cancer than in other non-metabolic diseases. Housing at TT has been shown to confer tumor-growth resistance in mice with various types of cancers (34, 54, 55). For example, mice inoculated with skin melanoma, colon carcinoma, pancreatic carcinoma, or mammary gland adenoma tumor-cells all had lower tumor formation, growth, and metastasis at TT compared to ST (54). In another study, mice inoculated with mammary gland adenoma had smaller tumors size at TT compared to ST (55). This effect was determined to involve a change in number, activity and function of CD8+ T cells and dendritic cells at higher temperatures (54, 55). A ST environment increases the number of immature or incomplete phenotype DCs, while at TT, CD8+ T cells are more likely to be activated and express IFNγ (54, 55). Also, mice housed at ST exhibited cold stress accompanied by elevated levels of NE that decreased the energy available for their immune system and reduced their response to tumor cells (4, 56, 58, 59, 76). Mohammadpour et al., showed that mammary gland adenoma murine models, circulating NE released under ST conditions activated MDSCs through their β-AR, resulting in decreased T cells proliferation and increased tumor growth (34). This effect was reversed, in β-AR knockout mice at ST or with mice treated with β-AR agonists at TT (34). The beneficial effect of TN housing was further demonstrated by the preference for that temperature in tumor-bearing mice when given the choice (54). The effect of cold stress at ST is even stronger in individually ventilated cages (IVC), a system that is widely used in the current laboratory settings (77). Mice housed in IVCs had smaller tumors, lower tumor metabolism, larger adrenal weights, and more signs of cold stress than those housed in a static system at the same temperature (77). This was somewhat reduced when the mice were given the opportunity to shelter with nesting material (77).

TN may have a positive influence on various cancer therapies. Mice with colon adenocarcinoma had lower responses to irradiation and chemotherapy at ST compared to TT (56). Mice bearing pancreatic tumors had higher sensitivity to cytotoxic treatments at TT compared to ST (25). Melanoma tumor-bearing mice had lower tumor growth after 3 weeks of aquatic exercise at TT compared to ST (57). This response was attributed to an enhanced cytotoxic-cell mediated immune response characterized by increases in populations of natural killer cells; natural killer T cells; γδT cells; CD8+ T cells; and, IFN-γ at the higher temperatures (57). In addition, resistance to treatments at ST was mostly attributed to elevated levels of NE because treatment with beta-blockers resulted in responses similar to those observed at TT (4, 25, 56, 58, 59, 76). Interestingly, higher levels of anti-apoptotic molecules (BAD, MCL-1, BCL-2, and BCLxL) and CREB, a transcription factor involved in the survival of these molecules were seen at ST compared to TT, which may also contributed to lower treatment efficacy at ST (25).

An irradiation-induced cancer model displayed a higher propensity for hematopoietic stem cells to undergo apoptosis when the animal received total body irradiation at TT compared to ST (78). Therefore, investigation of tumorigenesis in mouse models only at ST is likely to limit our understanding of this process.

The studies described above were limited to certain types of cancers and do not exclude that some cancer types would react differently to a TT environment. Also, different acclimatization times for the cage temperatures in the studies were likely to affect mouse metabolism differently (25, 54–56, 76, 78).

## Metabolic Diseases

Most studies conducted at TT have been on obesity-related research. Obesity can be induced in mice by providing them with an *ad libitum* high fat diet (HFD) (79). As mentioned in the introduction, the mouse metabolism is less active at TT with evidence of lower fasting glucose levels, decreased food intake, lower energy expenditure, and decreased BAT activation because the animal does not have to generate heat (21–24, 45). These diet-induced changes occur independently of the type of diet fed to mice (45). However, some diet-based differences occur at TT. In most HFD-obese mice models, mice housed at TT have lower glucose clearance, higher adiposity, increased hepatic fat accumulation, increased adipose inflammation, and greater glucose intolerance than mice at ST fed the same diet (45, 46). This suggests that housing at TT exacerbates the establishment of obesity.

Diet-dependent metabolic changes are closely related to the reduction of the NE-mediated BAT activation pathway at TT. Disruption of the pathway by gene knock-out or treatment with exogenous disruptors in mice housed at ST leads to similar results (29, 32, 49, 50, 80–82). For example, when C57BL6 mice were treated with BMP7, an inducer of BAT differentiation, they displayed increased food intake; increased BAT weights; increased expression of *ucp-1*, CD36 and lipase; increased energy expenditure; lower WAT weights, and higher lipolysis activity at ST, but not at TT (80). UCP-1 KO mice have been used to study the establishment of obesity because they were thought to be resistant to it when fed an HFD (50). However, it was shown that this resistance disappeared at TT because of a decrease in metabolic efficiency (81, 82). However, these KO mice did not exhibit adipose tissue inflammation at TT compared to ST, which suggests that BAT inflammation is due to exposure to cold and not to accumulation of fat (32). In addition, disruption of TH activity by knocking-out the type 2 deiodinase enzyme required for its activation had a similar effect as knocking-out UCP-1 (49). The obesity phenotype was decreased at ST, but increased at TT due to the inability of those mice to activate diet-induced thermogenesis compared to WT mice (49).

Thermoneutrality also has an effect on obesity-related disease presentations such as atherosclerosis and non-alcoholic fatty liver disease (NAFLD) (44, 51, 52, 83). Atherosclerosis is defined as plaque build-up coupled with immune cell infiltration in the blood vessel wall and is one of the leading causes of cardiovascular disease (CVD) (83). It occurs when there is an increase of lipids in the circulation as a result of changes in the metabolic environment (83). When housed at TT, mice display two metabolic markers of atherosclerosis (i.e., adipose and vasculature inflammation) faster than at ST, even if there is no change in insulin resistance (44, 51). Macrophage infiltration in the aorta and upregulation of pro-inflammatory cytokines, chemokines and other inflammatory mediators were observed at TT (51). In addition, the combination of a low metabolism at TN and a HFD initiated atherosclerosis in WT mice by altering blood lipid profiles and increasing aortic plaque size (83). In mice that are predisposed to atherosclerosis, the disease was exacerbated by the same combination (83). These results suggest that the link between adipose tissue inflammation and insulin-resistance only occur in cold stressed mice and thus are not applicable to our understanding of human atherosclerosis (51). Diet-induced NAFLD is the most common chronic liver disease and may lead to complications, the need for liver transplantation, and death (52). Female mice were thought to be resistant to the establishment of the disease due to hormonal differences (52). However, multiple strains of mice housed at TT and fed a HFD had lower corticosterone production; upregulated pro-inflammatory cytokines; and, exacerbated diet-induced NAFLD, independently of sex (52). Sex-independent increases in intestinal permeability and an altered microbiome were also observed at TT. Depletion of gram-negative bacteria, TLR4 deletion, or IL-17 axis activation reversed all TT-associated effects (52). This strongly implicates a role for environmental temperature on the composition of the gut microbiome in the establishment of NAFLD because the skewing of gram-negative bacteria at TT increases intestinal permeability, which in turn causes more LPS to interact with TLR4 to increase inflammation (52). This is particularly relevant because the small intestine is remodeled by the microbiome during cold stress acclimatization (53). These results led some researchers to speculate that the mouse microbiome is different at TT compared to ST, but more studies should be conducted to establish the effect of TN on the microbiome (8). Housing temperature does not seem to influence joint replacement therapy failure models (84). Joint replacement is used to treat joint degenerative disease. Wear and tear on the joint as well as lower circulating levels of leptin in obese individuals are thought to play a role on osteolysis leading to joint replacement failure (84). However, obese (*ob/ob*) mouse models are resistant to the development of osteolysis at ST and TT suggesting that this effect is independent of TN (84).

Finally, some of the proposed obesity treatments have different outcomes at TT compared to ST (47, 48). Because obese mice treated with DNP, a weight loss drug, had no reduction in obesity phenotype at ST, the drug was initially thought to be ineffective (47). However, although treatment with DNP for 4 weeks at TT did not affect food intake, energy expenditure increased; body weight and fat mass decreased; glucose tolerance improved; steatosis was reduced; and decreases were observed in TH levels, UCP-1 expression and BAT thermogenesis (47). In addition, when mice underwent weight reduction surgery, they showed similar energy reduction at ST and TT, but those at TT showed lower energy basal demand at TT (48). They also exhibited less torpor, defined as a depression in all physiological functions at TT, suggesting that TN improved the outcomes of surgery by enabling mice to recover better (48).

The limitations of these studies include different temperatures; mice models; and, acclimatization times used in different experiments. These difference may explain why some contradictory effects where observed in different studies such as the presence or absence of temperature-dependent glucose tolerance at TT (45, 46).

## Cardiovascular Diseases

There are clear links in rodents between metabolic activity and changes in cardiovascular system activity that can predispose to CVD. Several studies showed that the metabolism of rodents at TT changes some of their cardiovascular parameters (22, 85–87). Mice and rats housed at temperatures oscillating between ST and TT, had lower mean blood pressure; heart rate; and, pulse pressure when the cage temperature was closer to TT as a consequence of lower thermogenic demands at TT (11, 85). The differences were more significant in mice than rats, most likely due to their greater metabolic demands (69). Similar results were also obtained for mice and rats housed at TT compared to ST (22, 87). These changes are clearly representative of a change in metabolism since they were observed with greater intensity when mice at TT were fasting (86). Oxygen consumption was unaffected at TT for mice but decreased for rats (86). These changes can predispose rodents to cardiovascular events like coronary artery disease or atherosclerosis, described in the *Metabolic Diseases* section (44, 51). It is not yet clear whether TN has a protective effect on other CVDs.

## Alzheimer’s Disease

The impact of TN on the central nervous system has been studied in the context of Alzheimer’s disease (AD) (74). Vandal et al. found that the triple-transgenic mouse model of AD (3×Tg-AD) was more susceptible to a cold environment despite having a higher non-shivering thermogenesis activity than WT mice, thus suggesting that ST would play a greater impact on these mice (74). At TT, the memory function and neuropathology of 3xTg-AD mice was improved within a week compared to 3xTg-AD mice housed at ST (74).

## Opposing Views to the Thermoneutral Model

Most of the current literature on TN advocates for conducting experiments on mice in housing at approximately 30°C since it is within the TNZ of mice (1, 3–6, 8, 13, 58, 88, 89). Thus results obtained at this temperature are more likely to be consistent with the metabolic state experienced by humans (1, 3–6, 8, 13, 58, 88, 89). However, some investigators have argued that the state of mice at this temperature is not representative of the average human metabolic state and experiments conducted at 30°C cannot be extrapolated to humans (90). In their review, Speakman et al. state that most humans function in environments that are on average 3°C below their lower critical temperature (Tlc) (90). This means that they occupy a space at a temperature that is below the lowest temperature within their TNZ (90). This metabolic state in humans corresponds to about 23–25°C in mice (90). In addition, Speakman et al. argue that mice are routinely given the opportunity to shelter and huddle in group housing, which increases their BCT (90). In this context, the researchers claim that the Tlc of the mice is further reduced to 20–22°C (90). One of the assumptions of this argument is that the murine response to change in environmental temperature is the same as the human response (90). However, as we mentioned in the introductory section of this paper, the metabolism of mice and humans is very different. Mice have a higher metabolic demand due to their smaller SA/V ratio and they rely more heavily on non-shivering thermogenesis than humans (1, 5). In addition, there is substantial evidence that humans mostly operate at a temperature within their TNZ (3, 11, 13). Thus, we are in agreement with the belief held by other investigators that more murine studies should be conducted at TT and the results compared to those obtained at ST. This will provide a better understanding of mouse disease models and refine their use for application to human health.

## Conclusion

As outlined in the *Introduction*, very few results obtained in mouse studies translate to human trials (9, 12, 16). This is generally attributed to biological differences between mice and humans and has led to calls by the scientific community to improve the murine models used to study human diseases (9, 12). One major aspect of studies with murine models that has been overlooked is the housing environment. Besides the consequences due to social stressors and microbiome differences, housing temperature also plays a role in the response to immune challenge (12, 16). Sub-optimal housing temperatures routinely used in the laboratory setting induce metabolic activity for heat generation, reducing energy available for metabolism of other biological functions like the immune system (5). The differing metabolic states of mice housed at ST and TT are likely to complicate our understanding of disease in murine models (**Figure 2**) (5). In this review, we illustrated that housing at TN could either exacerbate or have protective effects in the context of bacterial, viral and parasitic infections, as well as in metabolic, cardiovascular and cancer diseases. We also showed the differential effect of TT compared to ST housing in Alzheimer’s disease, respiratory disease, and graft *vs*. host disease. Taking into consideration the various original research articles and independent reviews done on the subject, we feel that it is safe to assume that housing temperature can influence the outcome of studies on murine models of disease and therefore affect our understanding of human diseases, in spite of confounding factors linked to sheltering material and group “huddling” (17, 90). We conclude that there is an advantage to investigating the role of TN in various disease models and comparing results to those obtained at ST in an effort to improve our understanding of human diseases.

## Author Contributions

FV and MO discussed and prepared the plan of the review. FV did the majority of the literature search and wrote the first draft of the review. FV and MO developed and drew the figures and table together. FV and MO edited the text and the overall presentation of the review. All authors contributed to the article and approved the submitted version.

## Funding

Research in the MO laboratory is supported by grants from the Canadian Institute for Health Research (Grant #159765) and the Natural Science and Engineering Research Council of Canada (Grant RGPIN/03863-2017).

## Conflict of Interest

The authors declare that the research was conducted in the absence of any commercial or financial relationships that could be construed as a potential conflict of interest.
